# Does an Adjustable-Loop Device Loosen following ACL Reconstruction with a Hamstring Graft? A Retrospective Study with a Follow-Up of Two Years

**DOI:** 10.3390/jcm11133648

**Published:** 2022-06-24

**Authors:** Mohammad Jesan Khan, Naiyer Asif, Mohd Hadi Aziz, Ariz Raza, Shahzad Anwar, Shibili Nuhmani, Ahmad H. Alghadir, Masood Khan

**Affiliations:** 1 Department of Orthopaedic Surgery, Jawaharlal Nehru Medical College Hospital, Aligarh Muslim University, Aligarh 202002, Uttar Pradesh, India; mzkhan2k1@gmail.com (M.J.K.); naiyerasif@gmail.com (N.A.); mohdhadiaziz@gmail.com (M.H.A.); arizrazakhan@gmail.com (A.R.); 2 Department of TB and Chest, Jawaharlal Nehru Medical College Hospital, Aligarh Muslim University, Aligarh 202002, Uttar Pradesh, India; anwar.shahzad44@yahoo.com; 3 Department of Physical Therapy, College of Applied Medical Sciences, Imam Abdulrahman bin Faisal University, Dammam 34221, Saudi Arabia; snuhmani@iau.edu.sa; 4 Rehabilitation Research Chair, Department of Rehabilitation Sciences, College of Applied Medical Sciences, King Saud University, Riyadh 11433, Saudi Arabia; aalghadir@hotmail.com

**Keywords:** anterior cruciate ligament reconstruction, adjustable-loop device, hamstring graft

## Abstract

Arthroscopic anatomic anterior cruciate ligament reconstruction (ACLR) is the gold standard treatment for an ACL tear and requires the use of fixed or adjustable-loop devices to fix a femoral-side graft. Although the adjustable mechanism is designed to provide one-way tensioning, there is a concern that the adjustable loop will loosen and lengthen during cyclic loads, creating graft laxity. The present paper is a retrospective study of patients who underwent ACLR with the fixation of a hamstring graft with an adjustable loop on the femoral side from November 2016 to October 2018. The knee’s functional outcome was evaluated using an International Knee Documentation Committee (IKDC) score, Lysholm score, Lachman test, and pivot shift test. The patients were assessed preoperatively and finally postoperatively after two years of surgery. Thirty-two patients were analyzed. Significant improvement was obtained in the final clinical outcome of the patients. Twenty-seven patients (84.4%) were Lachman negative, and twenty-eight patients (87.5%) were pivot shift test negative, the mean Lysholm score was 96.91, and the IKDC score was 91.47 (*p* < 0.001). There was no infection, graft failure, or flexion restriction. Arthroscopic ACLR with an adjustable-loop suspensory device is a successful fixation method for femoral-side graft fixation and offers a similar functional outcome as with fixed-loop devices.

## 1. Introduction

An anterior cruciate ligament (ACL) injury is the most common major sports trauma to the knee, and the arthroscopic anatomical reconstruction of it is considered the gold standard treatment. Anterior cruciate ligament reconstruction (ACLR) is performed on individuals with functionally unstable knees to restore normal knee kinematics. The primary objective is to obtain a secure initial graft fixation for tendon–bone healing and graft integration into the tibial and femoral tunnels. In addition, the fixation should be able to withstand slip during cyclic-loading circumstances to prevent loosening in the early post-operative phase. Popular femoral graft-fixing methods are the use of compression (interference screws), transfixation pins, or cortical-suspension-loop device.

Although the best femoral fixation system is controversial, conventional fixed-loop cortical-suspension devices provide excellent and consistent results and are widely used around the world [1]. Fixed-loop devices offer good graft fixation in terms of graft slippage and offer adequate graft strength. The fixed-loop device is made up of a metallic button and a polyurethane ribbon. The external cortical bone provides support for the button, while the ribbon connects the graft to the button. EndoButton (EB) CL (Smith & Nephew Inc, Andover, Massachusetts, USA) is one such example of a fixed-suspensory-loop system (FDL). To flip the endobutton over the lateral femoral cortex over drilling is needed, but in doing this a portion of the tunnel is left empty of graft leading to graft motion and tunnel widening, which hampers graft assimilation [1]. Furthermore, the anatomical position of the tunnel leads to a small tunnel length and a small segment of graft in the bone [2]. To overcome these limitations, second-generation adjustable-loop devices (ALDs) were introduced (TightRope (TR) (Arthrex Inc, Naples, Florida, USA)), which is based on a finger-trap mechanism. They are more convenient to use in short femoral tunnels. They allow more grafts to be filled in the femoral tunnel and shorter graft lengths can also be used. Over drilling is not necessary since loop adjustment and tightening can be performed during the operative procedure with respect to the length of the height of the tunnel, thus reducing the femoral attic and reducing the risk of the “bungee cord effect” [1,3,4].

Although the adjustable mechanism is designed to provide one-way tensioning, there is a concern that the loop will loosen and lengthen during cyclic loads, creating graft laxity. Several recent biomechanical studies have shown considerable loosening of the adjustable-loop system under conditions of cyclic-loading stress compared to a fixed-loop device, which could affect clinical and radiological outcomes following ACLR [1,2,5,6,7]. The available body of literature indicates that there is still a scarcity of adequate data to conclude the possible loosening of adjustable-loop devices and their clinical outcomes [8]. The present study uses an adjustable-loop system to analyze the loosening of the ACLR and clinical results in terms of various scores and instability measurements.

## 2. Material and Methods

**Figure 1:**
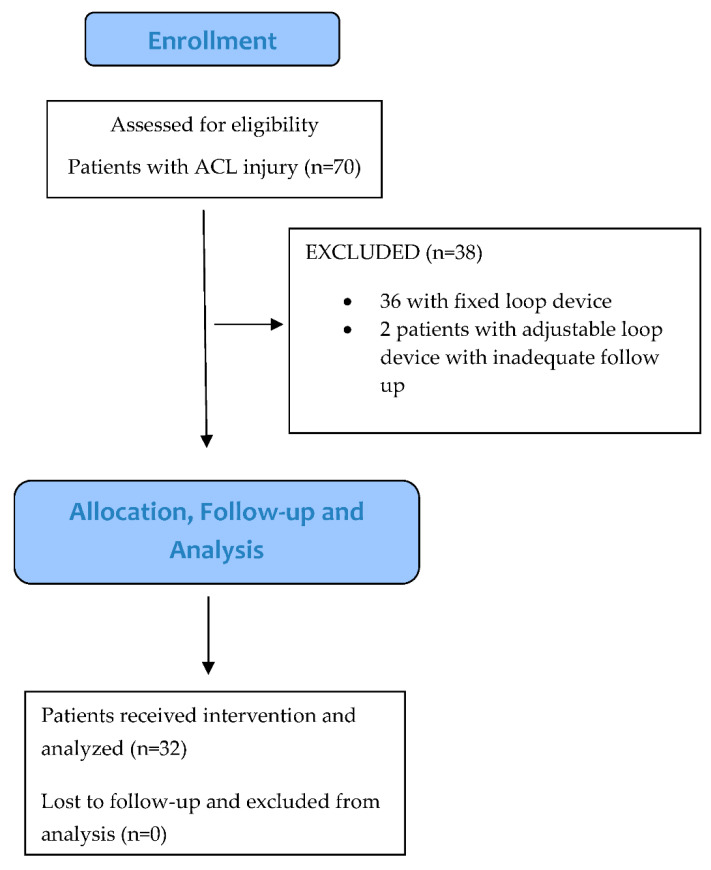
Flow chart showing recruitment, inclusion, and exclusion of participants.

This was a retrospective study on subjects with an ACL injury who visited our hospital from November 2016 to October 2018. Subjects in the age group of 18 to 50 years with an ACL tear or ACL tear with meniscal injury complaining of instability confirmed clinically and on MRI were included in the study. The exclusion criteria included patients operated with a fixed-loop device and patients with inadequate follow-up (Figure 1).

Thirty-two patients with an ACL injury were included. For femoral fixation, we employed an adjustable-length loop device TR (Tightrope, Arthrex Naples, FL, USA), and for tibial side-graft fixation, we used a bioabsorbable screw. The study was conducted in accordance with the Declaration of Helsinki, and approved by the Institutional Ethics Committee. All patients provided informed consent. A senior surgeon performed all the operations. Tourniquet was used in all the cases. The ipsilateral semitendinosus and gracilis tendons were harvested after confirming the diagnosis of ACL tear arthroscopically. In patients with a meniscal tear, partial meniscectomy was performed, and we did not perform the meniscal repair in any of the patients. After hyper-flexing the knee, a femoral tunnel was created through the anteromedial portal. A tibial tunnel was made with a tibial jig at 55°. The femoral tunnel was drilled according to the size of the quadrupled hamstring graft. TR was used for the fixation of the femoral side-graft and the bio-absorbable screw for the fixation of the graft to the tibia. Cycling was performed 20 times to remove any graft creep. A reassessment of the laxity of the graft was performed under arthroscopic visualization. Re-tensioning was performed until the ACL graft was completely tense and firmly placed in the femoral socket by alternate dragging of the white strands. Notch impingement and graft tightness were assessed by flexing and extending the knee. Three doses of antibiotic prophylaxis were administered during the perioperative period as per our institutional policy [9].

On the second postoperative day, full weight bearing with a crutch/walker was allowed. Patients were discharged on postoperative day three. Knee flexion was increased by 30° each week. A knee brace was advised for four weeks after surgery and then discontinued according to the patient’s comfort. After six to eight months, sports activities were allowed [10].

The functional outcome of the patients was evaluated using the International Knee Documentation Committee (IKDC) scoring method, Lysholm scoring method, Lachman test, and the pivot shift test [11,12,13]. We followed the patients for a minimum of two years after surgery. Data analysis was performed using SPSS software, version 20.0 (IBM Corp., Chicago, IL, USA). Chi-squared test was applied for categorical data(a), and for continuous data, the paired t-test(b) was applied. Sample-size calculation was performed with the help of the IKDC score, which was obtained from the study of Angthong et al. [14]. The mean preoperative and postoperative IKDC scores were 45.5 ± 13.8 and 58.6 ± 20.8, respectively. Taking these values as a reference, the sample size (80% power and 5% significance) was 15. Taking the loss to follow-up as 20%, the total sample size to be taken was 19.

## 3. Results

We evaluated the demographic information as well as the preoperative and postoperative functional scores (Table 1 and Table 2).

Lachman and pivot shift tests were performed on each patient preoperatively (Table 2). In the preoperative group, one patient (3.1%) was Lachman grade 1 positive, five patients (15.6%) were grade 2 positive, and 26 patients (81.3%) were grade 3 positive. The pivot shift test was grade 1 for 1 patient (3.1%) grade 2 for 22 patients (68.8%), and grade 3 for 9 patients (28.1%). The preoperative IKDC score was 55.8 ± 8.1 (40.2–66.7), and the Lysholm score was 73.0 ± 4.6 (65–80). The average wasting of the quadriceps muscle was 1.8 ± 0.8 cm (1.0–4.0).

After two years of surgery, we performed a final evaluation of the patients. There has been a significant improvement in the scoring and laxity assessments. There were five patients (15.6%) with positive Lachman grade 1 and four patients (12.5%) with positive pivot shift grade 1. The postoperative Lysholm score was 96.9 ± 1.69 (95–100) (<0.001) and the IKDC score was 91.4 ± 3.6 (87.4–96.6) (<0.001). The average wasting of quadriceps was reduced to 1.0 ± 0.5 cm (0.0–2.5) (<0.001).

There were no infections, graft failure, or flexion restrictions in any of the patients.

## 4. Discussion

Adjustable-loop devices are the newer version of femoral cortical suspension devices, offering multiple advantages. There is no need to pre-calculate the loop length, and over-drilling of the femoral tunnel is not required. Even in a short femoral tunnel, the maximum amount of graft can be pulled into the tunnel, avoiding multiple fixed-loop sizes in the inventory [8].

In our study, most of the patients required reconstruction as they were in grades 2 and 3 instability by the pivot shift and Lachman tests preoperatively. In most patients, we restored knee stability as 84.4 % tested negative for the Lachman test, and 87.5 percent of patients had a negative pivot shift test. Plaweski et al. [15] observed that 80.9 percent of patients with a fixed-loop device were pivot shift test negative. With a similar device, Ibrahim et al. [16] reported 86.6 percent of patients presenting negative for the Lachman test and 84.2 percent of patients presenting negative for the pivot shift test. Williams et al. [17] found that the Lachman and pivot shift tests were negative for 89% of patients who were fixed with an interference screw in the femoral tunnel. Shahpari et al. [18] found a negative Lachman test for 81.8% of the patients and a negative pivot shift test for 87% of the patients with a fixed-loop device.

In our study, a significant improvement in the functional results was observed in terms of the Lysholm (96.91) and the IKDC (91.47) scores. In the study conducted by Khan et al. [19], the mean Lysholm score was 92 and the mean IKDC score was 83 with a fixed-loop device. The study conducted by Plaweski et al. [15] revealed a Lysholm score of 94.1, whereas an IKDC score of 91.4 was revealed with a paired device. With a similar apparatus, Charlton et al. [20] observed a mean Lysholm score of 91 and a mean IKDC score of 83. Xu et al. [21] recorded a Lysholm score of 93 and an IKDC score of 95.7 in patients fixed with a fixed-loop device. Ibrahim et al. [16] and Williams et al. [17] discovered that the average Lysholm score was 92 and 91, respectively, with an analogous device. With a fixed-loop device, Shahpari et al. [18] obtained average Lysholm and IKDC scores of 90 and 85, respectively. Even though we used adjustable-loop devices in our patients, our findings were equivalent to those of fixed-loop devices.

Some in vitro experiments have suggested that an adjustable-loop device is inferior to a fixed-loop device, and raises concerns about the propensity of the former to elongate during cyclical loads, thus compromising functional graft length. This is crucial during the initial 8–12 weeks after surgery during the early recovery phase when the graft is healing. Lengthening in this phase results in functional knee instability due to impaired tendon–bone healing. Graft failure criteria are widely accepted as >3 mm elongation or side-to-side difference [22]. In one biomechanical study, researchers discovered that device slippage caused a higher displacement of the adjustable loop when the variable loop design was tested during cyclic and pull to failure loads [6]. In one study, during cyclic tests, researchers found clinically significant changes in loop length (>3 mm) due to the pulling of free suture ends into the adjustable loop [2]. Many other authors observed identical findings in their research [2,7]. In the displacement testing of individual devices, one author discovered a substantial variation in their displacements, but experimenting with pig bone and cattle tendon showed no noteworthy variation in displacements [1]. Furthermore, Glasbrenner et al. [23] also found a remarkable limitation in the finger-trap mechanism when subjected to complete unloading during cyclic testing. The loop was elongated to more than 3 mm after 200 loading cycles.

Despite all these facts, other researchers claim that there is no significant variation between the two devices [24,25]. In our study, the functional outcome in terms of IKDC and Lysholm scores and knee laxity assessment using the Lachman and pivot shift tests at the final follow-up were similar to the other studies on fixed-loop cortical-suspension devices. However, many biomechanical studies reveal lengthening in adjustable-loop systems with cyclic displacement, and they are inferior to fixed-loop systems.

The results of most biomechanical studies do not reflect clinical studies. This may be because laboratory tests do not provide a good enough simulation of the patient’s biomechanical and physiological fixation environment. Altered angles of ACL loading in vivo [2,5], the different bone mineral density of human bone as compared to porcine bone [26], and the effect of other confounding factors, such as graft healing, can be the probable cause [27,28,29,30]. In general, in vitro specimens are designed to mimic physiological loads, but the laboratory-based nature of the study limits their clinical applicability. Furthermore, the complicated force vectors generated in vivo by an ACL graft differ from those observed in vitro. In addition, the graft does not suffer enough force during the initial rehabilitation phase, leading to elongation. Furthermore, cycling the graft and securing it in tension on the tibial side reduced the effects of elongation during the initial cycles [31].

The present study had several limitations. To begin with, rather than using an instrument-assisted knee laxity testing (e.g., arthrometer) method, subjective evaluations were employed to determine knee stability. We used the International Knee Documentation Committee (IKDC) scoring method, Lysholm scoring method, Lachman test, and the pivot shift test for the outcome measures. In our study, all the measurements were performed by a senior professor in the department, having clinical experience of more than 20 years, who was blinded to the type of surgery. Therefore, the subjective variations were minimized. The validity and reliability of these tests have been confirmed in previous studies [32]. Furthermore, few authors have given equal weight to the Lachman test and KT 1000 arthrometer [33]. Moreover, Konig et al. [34] did not advocate the use of instruments for assessing knee laxity after assessing the endpoint of the Lachman test. On the contrary, the reliability of the instrument-assisted knee laxity test has also been questioned in previous studies [35] and there is still a debate on which instrument has to be used for this measurement. Secondly, we did not consider tunnel widening. Thirdly, the study included a retrospective review of patient data that had been obtained prospectively. Lastly, the sample size was small, and the follow-up period was short.

## 5. Conclusions

In patients with an ACL injury, arthroscopic anatomic reconstruction of the ACL with an adjustable-loop suspensory device is an efficient fixation technique for the attachment of the graft to the femur. It produces comparable outcomes in terms of knee stability, IKDC score, and Lysholm score. Despite biomechanical studies showing graft slippage with these devices, our study revealed that using an adjustable-loop device to attach the graft to the femur during ACLR is an efficient method. To confirm our results, more studies are needed using randomization between fixed and adjustable-loop devices, large sample sizes, and longer follow-ups.

## Figures and Tables

**Table 1:** Demographic properties of the participants involved in the study.

Characteristics	Mean	Number
Gender		
Male (number)		29
Female (number)		03
Age (years)	26.0 ± 7.2 (18–43)	
Time since the injury to surgery (months)	10.3 ± 4.3 (3–18)	
Side involved		
Right (number)		21 (65.6%)
Left (number)		11 (34.4%)
Medial meniscus tear (number of subjects)		10 (31.2%)
Lateral meniscus tear (number of subjects)		4 (12.5%)
Femoral tunnel length (mm)	39.5 ± 3.8 (33–45)	
Graft diameter (mm)	8.4 ± 0.64 (7.0–9.5)	
Quadrupled graft (mm)	90.8 ± 8.1 (80–110)	
Follow-up period (months)	34.2 ± 5.4 (26–44)	
Mechanism of injury (number of subjects)		
Sports	20	
RTA	08	
Injury during daily routine activities	04	

**Table 2:** Functional assessment of participants. Results are presented as numbers (percentage).

	Pre-Operative	Post-Operative	*p*-Value
**Lachman Test**			<0.001 ^a,^*
Grade 0	0 (0%)	27 (84.4%)	
Grade 1	1 (3.1%)	5 (15.6%)	
Grade 2	5 (15.6%)	0	
Grade 3	26 (81.3%)	0	
**Pivot Shift Test**			0.003 ^a,^*
Grade 0	0 (0%)	28 (87.5%)	
Grade 1	1 (3.1%)	4 (12.5%)	
Grade 2	22 (68.8%)	0	
Grade 3	9 (28.1%)	0	
IKDC Score	55.8 ± 8.1 (40.2–66.7)	91.4 ± 3.6 (87.4–96.6)	<0.001 ^b,^*
Lysholm Score	73.0 ± 4.6 (65–80)	96.9 ± 1.69 (95–100)	<0.001 ^b,^*
Thigh Circumference (cm)	1.8 ± 0.9 (1.0–4.0)	1.0 ± 0.5 (0–2.5)	<0.001 ^b^

* Significant. ^a^ = Chi-squared test, ^b^ = Paired *t*-test, IKDC = International Knee Documentation Committee.

## Data Availability

The data associated with the paper are not publicly available, but are available from the corresponding author upon reasonable request.
